# A Rare Case of a Giant Parathyroid Adenoma in a Young Male Patient

**DOI:** 10.7759/cureus.81911

**Published:** 2025-04-08

**Authors:** Sheon Baby, Natalia Pereira, Shirley Kim

**Affiliations:** 1 Internal Medicine, University of Florida Health, Gainesville, USA

**Keywords:** adult primary hyperparathyroidism, giant parathyroid adenoma, humeral fracture, hungry bone syndrome, parathyroid gland adenoma

## Abstract

A giant parathyroid adenoma (GPTA) is a rare type of primary hyperparathyroidism (PHPT) characterized by a weight of at least 3.5 g. We report a case of a 24-year-old male patient who presented with generalized fatigue and pathological left humeral fracture, found to have a serum calcium level of 16.9 mg/dL and a parathyroid hormone (PTH) level of 3,164 pg/mL. A 5.4 g right inferior type E parathyroid adenoma was excised with normalization of PTH levels. His clinical course was complicated by hungry bone syndrome and required management by a multidisciplinary team involving internal medicine, endocrinology, endocrine surgery, orthopedic surgery, nephrology, thoracic surgery, and genetics.

## Introduction

Primary hyperparathyroidism (PHPT) is prevalent in 0.1% to 1.0% of the population, making it one of the most common endocrine disorders [1]. In the United States, it is two to three times more prevalent in women than in men and increases with age [2]. Primary hyperparathyroidism is characterized by elevated levels of parathyroid hormone (PTH) leading to hypercalcemia and hypophosphatemia. Primary hyperparathyroidism is most commonly caused by a single benign parathyroid adenoma (85%), followed by multiple parathyroid gland involvement, such as in hyperplasia or less frequently by adenomas (15%), and rarely by parathyroid carcinoma (1%) [3]. The normal parathyroid gland weighs approximately 50-70 mg, and parathyroid adenomas weigh less than 1 gram [4]. A giant parathyroid adenoma (GPTA) is extremely rare and is generally defined as weighing at least 3.5 g [5]. Our case reports a young male patient found to have a 5.4 g parathyroid adenoma.

## Case presentation

**Figure 1:**
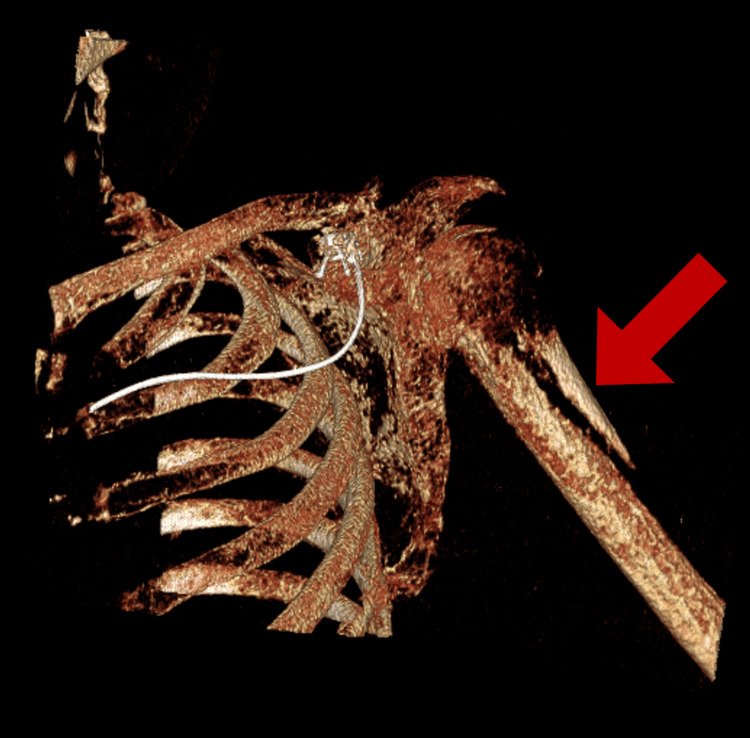
Left proximal humerus pathologic fracture (arrow)

**Figure 2:**
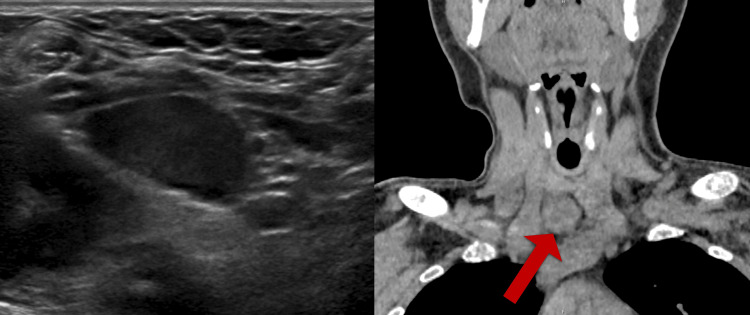
Thyroid ultrasound (left) and CT of the parathyroid scan (right; arrow) revealing right inferior thyroid nodule

A previously healthy 24-year-old male patient presented with fatigue and an atraumatic left humeral fracture. He was found to have a calcium level of 16.9 mg/dL and a PTH level of 3,164 pg/mL. A CT of the left upper extremity demonstrated an oblique left proximal humerus pathologic fracture (Figure 1) and diffuse bony demineralization. A lytic lesion of the left humeral head and an additional lytic lesion of the left lateral sixth rib were also identified. Thyroid ultrasound revealed a 2.9 cm hypoechoic right inferior thyroid nodule, which was confirmed on the CT scan to be a distinct lesion as opposed to exophytic from the thyroid, raising concern for a parathyroid adenoma (Figure 2). The patient’s hypercalcemia was medically managed with IV fluids, calcitonin, and zoledronic acid.

**Figure 3:**
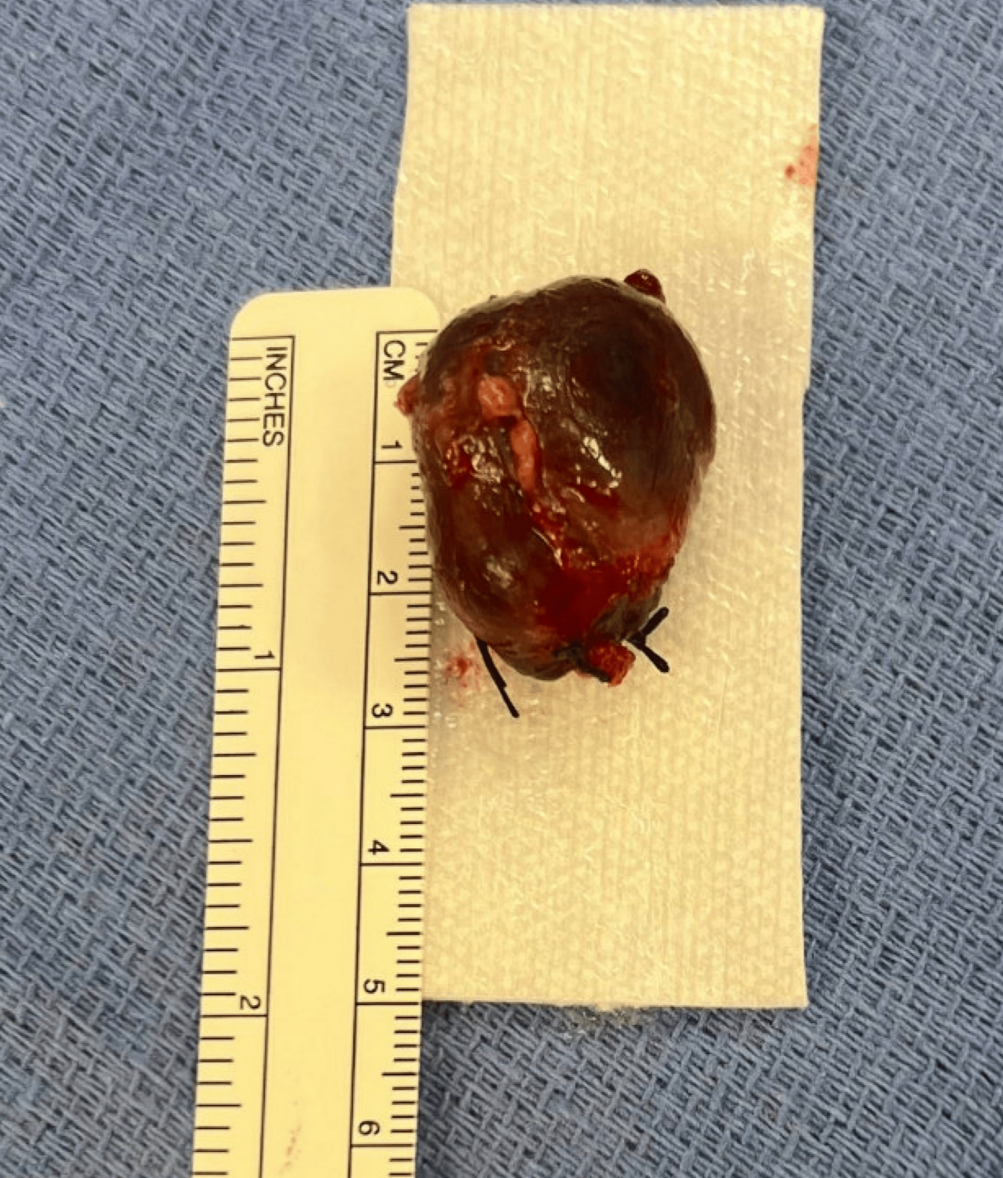
Giant parathyroid adenoma weighing 5.4 grams

Endocrine surgery evaluated the patient, and he was found to meet criteria for parathyroid removal given serum calcium and age <50 years [3] in the setting of a pathologic fracture. An incision was made above the clavicle in the midline, and a right-sided inferior type E parathyroid adenoma that was soft and dark red in color was identified (Figure 3). Intraoperative PTH (ioPTH) monitoring revealed a significant decline in PTH from the initial pre-excision level of 3,526 to 304 pg/mL 10 minutes after excision. Given the patient’s age, family history, and absence of genetic testing, bilateral cervical exploration was performed, which revealed that the remaining three parathyroid glands were small and diminutive. The ioPTH level after bilateral cervical exploration measured 207 pg/mL and, 30 minutes later, measured 139 pg/mL. Due to the lack of normalization of PTH, there was concern for persistent disease. As such, the carotid sheath and paraesophageal areas were skeletonized bilaterally, but no additional parathyroid gland was identified. Bilateral cervical thymectomies were performed for the possibility of supernumerary parathyroid glands. After cervical thymectomy, the ioPTH level normalized to 84 pg/mL. Pathology confirmed a hypercellular enlarged parathyroid gland weighing 5.4 g, seven benign lymph nodes, and benign bilateral thymic tissue.

**Figure 4:**
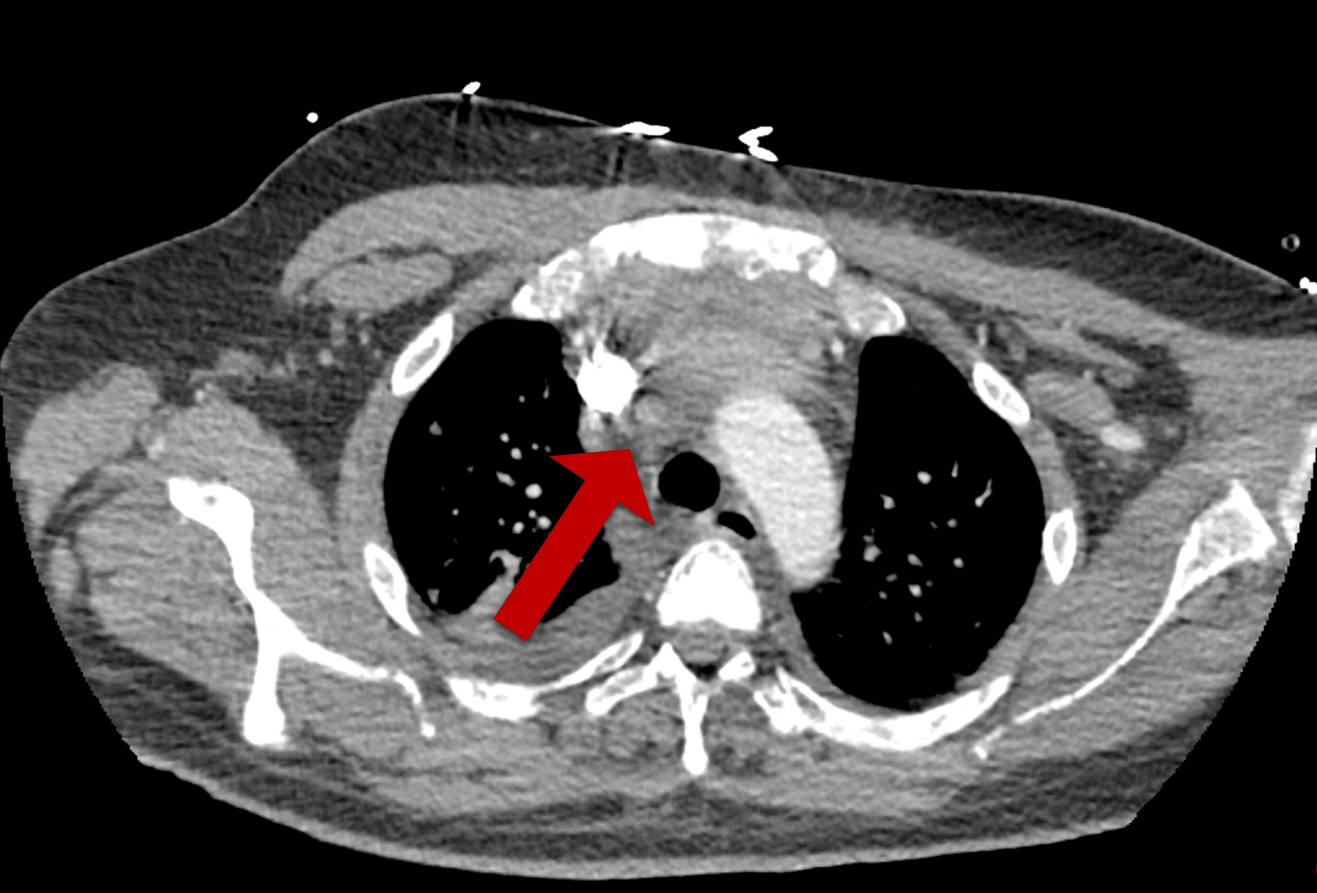
Stable mediastinal hematoma on the CT scan (arrow)

The patient’s postoperative course was complicated by severe hungry bone syndrome, for which he was admitted to the ICU for frequent electrolyte monitoring and calcium infusion. The patient was started on normal saline with calcium gluconate infusion at 21 mL/hr for one day and then transitioned to 10 mL/hr for two days. Simultaneously, he was supplemented with calcitriol 0.5 mcg bid and calcium carbonate 3 g three times a day (tid). The patient also suffered from acute blood loss anemia, which required transfusion of three units of packed red blood cells (pRBCs). A CT scan revealed a mediastinal hematoma (Figure 4) without active hemorrhage. Thoracic surgery evaluated the patient, and he did not require surgical interventions. Furthermore, his electrolytes showed metabolic acidosis with a bicarbonate level of 14 mmol/L, and he had a worsening creatinine from 1.8 mg/dL on admission. Nephrology evaluated the patient, and since his baseline creatinine was unknown, it was determined that his elevated creatinine of 2.4-2.5 mg/dL was likely his new baseline. The management strategy consisted of treating his underlying hypercalcemia with hope for renal recovery in the future. Ultimately, the patient was weaned from the calcium infusion and discharged with calcitriol 2 mcg two times a day (bid) and calcium carbonate 7.5 g every six hours (q6h). Multidisciplinary follow-ups with endocrinology, endocrine surgery, orthopedic surgery, nephrology, and genetics were arranged.

## Discussion

We report a case of a 24-year-old male patient who presented with a nontraumatic humeral fracture and generalized fatigue and was found to have a calcium level of 16.9 mg/dL, PTH level of 3,164 pg/mL, and a 2.9 cm right inferior thyroid nodule. He underwent surgical excision, which revealed a 5.4 g giant parathyroid adenoma. Although parathyroid adenomas are the most common cause of PHPT, a GPTA is extremely rare [3]. A systematic review and meta-analysis published in 2022 has reported only 65 cases of giant parathyroid adenomas [6]. The median age was 53 years, with the youngest being a 12-year-old female patient and the oldest being an 85-year-old female patient, with the majority being female (68%) [6]. The largest GPTA was reported in 2011 and weighed 145 grams with an associated PTH level of 642 mg/mL; however, the patient reportedly had minimal symptoms [7]. Interestingly, the study found no correlation between the sizes of GPTAs and PTH, nor between their weights.

Clinical manifestations of GPTAs include fatigue, bone pain, pathological fracture, nausea/vomiting, renal colic, depression or anxiety, and abdominal pain, as well as a palpable neck mass [7]. Diagnosis of PHPT begins with a detailed history with an emphasis on family history for hereditary conditions such as MEN1 and 2, as well as a drug history with an emphasis on diuretics and psychiatric medications such as lithium. Our patient was previously healthy and did not take any medications. Family history was significant for a mother with primary hyperparathyroidism at age 45, with the removal of one enlarged parathyroid gland and one ectopic gland. However, she did not have a history of pituitary or adrenal abnormalities, making MEN syndromes less likely. Our patient was referred to the genetics outpatient for both testing and counseling.

Hungry bone syndrome refers to the profound (serum calcium <2.1 mmol/l) and prolonged (longer than four days postoperatively) hypocalcemia that follows parathyroidectomy for severe hyperparathyroidism [8]. Severe hypocalcemia results from an increased influx of calcium into the bone due to the sudden removal of high levels of PTH, causing bone resorption [8]. Risk factors for hungry bone syndrome include older age, weight/volume of the resected parathyroid glands, vitamin D deficiency, and radiological evidence of bone disease [8]. Our patient had evidence of brown tumors, or osteitis fibrosa cystica, on CT characterized by two lytic lesions and diffuse bony demineralization. His parathyroidectomy was complicated by hungry bone syndrome, requiring calcium gluconate infusion and oral calcium supplementation.

## Conclusions

A GPTA is a rare type of PHPT. We hope our case report adds to the literature on the diagnosis and management of GPTAs. To provide holistic care to these patients, a multidisciplinary team involving internal medicine, endocrinology, endocrine surgery, orthopedic surgery, nephrology, thoracic surgery, and genetics is often necessary.

## References

[1] Kowalski GJ, Buła G, Żądło D, Gawrychowska A, Gawrychowski J (2020). Primary hyperparathyroidism. Endokrynol Pol.

[2] Yeh MW, Ituarte PH, Zhou HC (2013). Incidence and prevalence of primary hyperparathyroidism in a racially mixed population. J Clin Endocrinol Metab.

[3] Bilezikian JP, Khan AA, Silverberg SJ (2022). Evaluation and management of primary hyperparathyroidism: summary statement and guidelines from the Fifth International Workshop. J Bone Miner Res.

[4] Al-Hassan MS, Mekhaimar M, El Ansari W, Darweesh A, Abdelaal A (2019). Giant parathyroid adenoma: a case report and review of the literature. J Med Case Rep.

[5] Ghemigian A, Trandafir AI, Petrova E (2022). Primary hyperparathyroidism-related giant parathyroid adenoma (review). Exp Ther Med.

[6] Wong HK, Shipman K, Allan K, Ghabbour A, Borumandi F (2022). Giant parathyroid tumours in primary hyperparathyroidism: a systematic review. Langenbecks Arch Surg.

[7] Cakmak H, Tokat AO, Karasu S, Özkan M (2011). Giant mediastinal parathyroid adenoma. Tuberk Toraks.

[8] Witteveen JE, van Thiel S, Romijn JA, Hamdy NA (2013). Hungry bone syndrome: still a challenge in the post-operative management of primary hyperparathyroidism: a systematic review of the literature. Eur J Endocrinol.

